# Notch1 Is a 5-Fluorouracil Resistant and Poor Survival Marker in Human Esophagus Squamous Cell Carcinomas

**DOI:** 10.1371/journal.pone.0056141

**Published:** 2013-02-07

**Authors:** Jian Liu, Huijie Fan, Yuanyuan Ma, Dongming Liang, Ruixia Huang, Junsheng Wang, Fuyou Zhou, Quancheng Kan, Liang Ming, Huixiang Li, Karl-Erik Giercksky, Jahn Martin Nesland, Zhenhe Suo

**Affiliations:** 1 Department of Pathology, The First Teaching Hospital of Zhengzhou University, Basic Medical College, Zhengzhou University, Zhengzhou, Henan Province, China; 2 Department of Pathology, the Norwegian Radium Hospital, Oslo University Hospital, University of Oslo, Oslo, Norway; 3 Department of Oncology, The First Teaching Hospital of Zhengzhou University, Zhengzhou, Henan Province, China; 4 Department of Pathology, Institute of Clinical Medicine, Faculty of Medicine, University of Oslo, Oslo, Norway; 5 Department of Oncology, Anyang Tumor Hospital, Anyang, Henan Province, China; 6 Department of Surgery, Anyang Tumor Hospital, Anyang, Henan Province, China; 7 Department of Pharmacology, The First Teaching Hospital of Zhengzhou University, Zhengzhou, Henan Province, China; 8 Department of Medical Laboratory, The First Teaching Hospital of Zhengzhou University, Zhengzhou, Henan Province, China; 9 Department of Surgery, Institute for Cancer Research, the Norwegian Radium Hospital, Oslo University Hospital, University of Oslo, Oslo, Norway; National Cancer Center, Japan

## Abstract

Notch signaling involves the processes that govern cell proliferation, cell fate decision, cell differentiation and stem cell maintenance. Due to its fundamental role in stem cells, it has been speculated during the recent years that Notch family may have critical functions in cancer stem cells or cancer cells with a stem cell phenotype, therefore playing an important role in the process of oncogenesis. In this study, expression of Notch family in KYSE70, KYSE140 and KYSE450 squamous esophageal cancer cell lines and virus transformed squamous esophageal epithelial cell line Het-1A was examined by quantitative RT-PCR. Compared to the Het-1A cells, higher levels of Nocth1 and Notch3 expression in the cancer cell lines were identified. Due to the finding that NOTCH3 mainly mediates squamous cell differentiation, NOTCH1 expression was further studied in these cell lines. By Western blot analyses, the KYSE70 cell line which derived from a poorly differentiated tumor highly expressed Notch1, and the Notch1 expression in this cell line was hypoxia inducible, while the KYSE450 cell line which derived from a well differentiated tumor was always negative for Notch1, even in hypoxia. Additional studies demonstrated that the KYSE70 cell line was more 5-FU resistant than the KYSE450 cell line and such 5-FU resistance is correlated to Notch1 expression verified by Notch1 knockdown experiments. In clinical samples, Notch1 protein expression was detected in the basal cells of human esophagus epithelia, and its expression in squamous cell carcinomas was significantly associated with higher pathological grade and shorter overall survival. We conclude that Notch1 expression is associated with cell aggressiveness and 5-FU drug resistance in human esophageal squamous cell carcinoma cell lines *in vitro* and is significantly associated with a poor survival in human esophageal squamous cell carcinomas.

## Introduction

The Notch pathway is evolutionarily conserved with an important role in the processes such as cell proliferation, cell fate decision, differentiation and stem cell maintenance. Due to its fundamental role in stem cells[1], it has been speculated during the recent years that Notch family may have critical functions in cancer stem cells or cancer cells with a stem cell phenotype, therefore playing an important role in the process of epithelial-mesenchymal transition (EMT)[2]. In addition, targeting Notch has been considered as a novel strategy in cancer campaign[3].

Altered Notch signaling has been associated with different malignancies including pancreatic, breast and colon carcinomas, in addition to glioma, leukemia and lymphoma[4], [5]. Experimental evidence supports the notion that Notch can act both as an oncogene and tumor suppressor gene depending on its expression levels and timing in a cell-type and context-dependent manner. In studies of stem and/or progenitor cells isolated from the mammary gland [6], Notch pathway has been implicated in self-renewal of stem cells, maintaining stem cell potential and inhibition of differentiation. In line with these findings, the Notch function in promoting carcinogenesis has been reported. For example overexpression of activated murine Notch1 and Notch3 in transgenic mice blocks mammary gland development and induces mammary tumors [7].

Hes-1, the downstream molecule of the Notch pathway, has been associated with invasive and metastatic potential of osteosarcomas, and inhibition of Notch pathway by γ-secretase inhibitors could eliminate invasion in Matrigel without affecting cell proliferation, survival or anchorage-independent growth [8], [9]. Significantly high Notch1 expression was found in colorectal cancer cells compared with that of normal colorectal epithelial cells. Notch1 receptor and Hes-1expressions are reported to be upregulated along with colon cancer progression and chemotherapy resistance [10]. In another *in vitro* study of HK-2 cells data show that Notch signaling is required to convert the hypoxic stimulus into epithelialmesenchymal transition (EMT), increased motility, and invasiveness. Inhibition of Notch signaling abrogates hypoxia-induced EMT and invasion, and, conversely, an activated form of Notch can substitute for hypoxia to induce these processes [11].

But, in other contexts such as primary epithelial cells (keratinocytes), increased Notch activity may cause exit from the cell cycle and/or commitment to differentiation [12], [13]. In supporting such assumption, it has been reported that the expression of Notch1 is markedly reduced or absent in invasive cervical cancers [14]. Further study shows that the expression of activated Notch1 causes strong growth inhibition of HPV-positive, but not HPV-negative, cervical carcinoma cells. Increased Notch1 signaling causes a dramatic down-modulation of HPV-driven transcription of the E6/E7 viral genes, indicating a protective effect against HPV-induced transformation through suppression of E6/E7 expression [14]. In addition, aberrant notch expressions were also reported in human lung squamous cell carcinomas [15], [16].

In esophagus, *in vitro* study in a commercial esophagus squamous cell carcinoma cell line with a pcNICD expression vector indicates that activated Notch1 signaling pathway gave rise to proliferation suppression of the cells, accompanied with a cell cycle inhibition at the G0/G1 phase and apoptosis[17]. While supporting these observations, Notch1 gene expression and activity have been shown substantially down-modulated in squamous cancer cell lines and tumors, and studies in different cells and tissues reveal important crosstalk of Notch and P53[18]. Genetic suppression of Notch signaling in primary human keratinocytes is sufficient, together with activated *ras*, to cause aggressive squamous cell carcinoma formation, leading to a conclusion that Notch1 gene is a p53 target with a role in human tumor suppression through negative regulation of Rho effectors [19]. Furthermore, Notch mutation study of head and neck squamous cell carcinomas also suggests that Notch1 may function as a tumor suppressor gene rather than an oncogene in this tumor [20].

Although the function of Notch3 is highly indicated to squamous cell differentiation[21], [22], studies of Notch1function in response to hypoxia in squamous cell carcinoma cell lines and large series of clinicopathological correlation of Notch1 in human squamous cell carcinomas are still missing. In this study we intended to firstly assess the Notch family expression in three squamous esophageal cancer cell lines and a virus transformed squamous esophageal epithelial cell line, so that the most differentially expressed Notch protein(s) in the cancer and virus transformed cell lines could be identified for further functional and clinicopathological studies, in order to better understand their clinical correlation in a series of 156 patients with ten-year follow-up.

## Materials and Methods

### Cell lines and culture

The human esophageal squamous cell cancer cell lines KYSE70, KYSE140 and KYSE450 and virus transformed human normal esophageal squamous epithelial cell line Het-1A were purchased from the German Collection of Microorganisms and Cell Cultures (DSMZ, Germany) and maintained in 5% CO2 in RPMI 1640 medium supplemented with 10% fetal bovine serum and 100 U/ml penicillin G and 100 µg/ml streptomycin at 37°C with saturated moisture.

### Quantitative RT-PCR

Cells in 80% confluent in culture were collected for quantitative RT-PCR analyses of Notch family members. Total RNA was prepared using the RNeasy Micro Kit (QIAGEN, Cat#: 74004) and converted into double-stranded cDNA with 0.5 µg RNA into a 10 µl total volume using the RT^2^ First Strand Kit (QIAGEN, Cat. No. 330401). Quantitative real time PCR was carried out by ABI 7900 HT machine using the RT^2^ Profiler PCR Array Human Notch Signaling Pathway kit (QIAGEN, Cat. No. PAHS-059ZA). The thermal cycling conditions were 95°C for 10 minutes, followed by 40 cycles of 95°C for 15 seconds, and 60°C for 1 minute. To verify the NOTCH1 sequence, conventional RT-PCR with two primer pairs which were used in earlier studies and sequencing of the PCR product were performed since information for primer sequences from the QIAGEN kit was not available. The forward and reward Notch1 primers for the first primer pair (a) are 5′-GGGTCCACCAGTTTGAATGG-3′ and 5′-GTTTGCTGGCTGCAGGTTCT-3′, respectively, giving product of 306 bp [23]. The forward and reward Notch1 primers for the second primer pair (b) are 5′-CTACCTGTCA GACGTGGCCT-3′ and 5′-CGCAGA GGGTTGTATTGGTT-3′, respectively, giving a product of 357bp [24]. Sequencing of both forward and reward PCR products was performed with BigDye Terminator v1.1 Cycle Sequencing Kit and and analyzed with ABI PRISM 3130 Genetic Analyzer (Applied Biosystems) after the PCR products were purified with BigDye XTerminator Purification Kit (GE Healthcare Life Science, Uppsala, Sweden).

### Western blot analysis

The Western blotting procedure was published before [18]. Membranes were blocked with 5% non-fat dry milk in TBST for 60 minutes and incubated with the primary antibodies at optimal dilution in TBST/2.5% milk overnight at 4 °C, i.e. goat anti-GAPDH (0.2 µg/ml, R&D, UK), mouse anti-Oct3/4 (1 µg/ml R&D, UK), mouse anti-Sox2 (1 µg/ml R&D, UK), HIF-1α (1 µg/ml R&D, UK), HIF-2α (1 µg/ml R&D, UK), rabbit anti-Notch1 (1 µg/ml, Cell Signalling, UK) and mouse monoclonal antiHes-1 (1 µg/ml, Abcam, UK). The membranes were then incubated with corresponding secondary HRP-conjugated antibodies before the immuno-complexes were visualized by enhanced chemiluminescence (GE Healthcare UK). The western blotting experiments were repeated at least three times.

### Hypoxic cell culture

After 24 hours cultivation in conventional cell culture in normoxia (20% O2) as described above (allowing cells to attach) the cell culture flasks were transferred into 1% hypoxic incubator for hypoxic cell culture experiments. The Xvivo Closed Incubation System (Xvivo system 300C, Biospherix, USA) was used in this study to obtain accurate oxygen tension in different incubators. Three flasks of the cells in each oxygen concentration group (20% and 1%) were harvested after variable time intervals of incubation, in order for further cell growth curve, Western blotting and drug sensitivity analyses.

### Cell proliferation analysis and chemosensitivity examination

Cell proliferation was measured by the 3-(4, 5-dimethylthiazol-2-yl)-2,5diphenyltetrazolium bromide (MTT) rapid colorimetric assay (Sigma-Aldrich). The KYSE70 and KYSE450 cells were seeded onto 96-well plates (5000/well with 180 µl RPMI 1640 medium supplemented with 10% fetal bovine serum) for variable time periods of culture before 20 µl of MTT solution (0.5 mg/ml) was added for additional 4 hours incubation before 100 µl dimethyl sulfoxide (Sigma-Aldrich) was added for 15 minutes. The plates were then shaken at low speed for 5 min and measured at 570 nm using a spectrophotometer. Triplicate wells were assayed for each condition and S.D was determined. Growth inhibitory rate was calculated as follows: (average OD value in the control group − average OD value in the treatment group)/average OD value in the control group×100%.

The dose dependent effect of 5-FU on cell proliferation was assayed by the MTT method as described above. Briefly, the cells with or without 5-FU (5-fluorouracil, Sigma-Aldrich) in the RPMI 1640 medium supplemented with 10% fetal bovine serum were cultured for 48 hrs before the cells were harvested for MTT analyses.

### Notch1 siRNA knockdown

KYSE70 cells were transfected with the siRNA against Notch-1 (sc-36095, Santa Cruz Biotechnology, Santa Cruz, CA) using Oligofactamine (Invitrogen) according to the manufacturer's instructions, with non-specific siRNA (sc-44236, Santa Cruz Biotechnology, Santa Cruz, CA) as control. 5 hrs after transfection the medium containing transfection complexes was replaced with fresh medium and the cells were incubated for another 48 hrs. The cells were harvested, and cell lysates were prepared for Western blotting as described above. To determine 5-FU sensitivity difference in the KYSE70 esophageal cancer cells in which Notch1 expression was confirmed to be blocked by gene knockdown, additional MTT analyses were performed on these cells with the method as described above.

### Patients and materials

In total, one hundred and fifty-seven patients, 95 men and 62 women, who underwent potentially curative surgery with diagnosis of esophageal squamous cell carcinoma during the period of 1989–1994 at the Anyang Tumor Hospital, Henan, China, were enrolled in this retrospective study. The one hundred and fifty-seven surgically-removed specimens were routinely fixed in formalin, processed and embedded in paraffin block for diagnosis and research use. In additional, 10 normal specimens adjacent to tumor were also included in this study. This project was approved by Anyang Hygiene Bureau and Anyang Tumor Hospital for the Sino-Norwegian collaboration project [25]. All the patients gave written consensus for this research application and all the written consents were filed in Anyang Tumor Hospital, Henan, china.

### Immunohistochemical method

Tissue microarray sections were applied in this study for screening of protein expression. Multi-tissue microarray blocks were prepared by using MTA-1 manual tissue arrayer (Beecher Instruments Inc., Sun Prairie, WI, U.S.A). Firstly, Hematoxyline and Eosin (H&E) staining sections made from the paraffin blocks were used to define two representative tumor areas and one stroma area. Secondly, the defined regions on paraffin block were transferred by a hollow needle, with cores diameter of 0.6 mm, to a recipient paraffin block. Finally, 3 µm sections from these recipient paraffin blocks were cut and mounted on charged Super-Frost Plus glass slides for immunohistochemistry analysis before being dried at 60°C in an oven for 24 hours. To improve the quality of the immunohistochemistry, whole tissue sections from these samples were also repeatedly stained with the same immunohistochemical procedure as for the tissue microarray sections.

For immunohistochemical staining Dako EnVision^TM^ + System, Peroxidase (DAB) (K4007, Dako Corporation, CA, and U.S.A) was employed. The sections were deparaffinized in xylene, microwaved in 10 mM citrate buffer pH 6.0 to unmask the epitopes, and treated with 0.3% hydrogen peroxidase (H2O2) for 5 min to block endogenous peroxidase. The Notch1 (d1E11) XP^TM^ Rabbit mAb (Cell Signaling, UK) and HES1 mouse monoclonal antibody (ab87395, Abcam, UK) were used in this immunohistochemistry according to the venders' instructions. After rinsed with DAKO wash buffer the sections were incubated with hydrogen peroxide for 5 minutes, and then incubated with primary antibody for 30 minutes at room temperature. After another rinse with DAKO wash buffer, mouse/rabbit EnVision FLEX+Linker reagent was added and samples were incubated for 15 minutes at room temperature, followed by incubation with EnVision FLEX+HRP for 30 minutes at room temperature. The sections were rinsed, color reaction developed with DAB reagent, counterstained in hematoxylin for 20 seconds, dehydrated, and mounted under glass cover slips in preparation for evaluation under microscopy.

Immunostaining was scored. Only immunoreactive intensity was considered since tumor cells, if were positive, were rather homogeneously stained. The tumors were scored as negative if no positive tumor cells were observed, and 1 was scored if the tumor cells were weakly immunoreactive, 2 was scored if the tumor cells were moderately positive and 3 was scored if the tumor cells were strongly positive.

### Statistical analyses

The associations between expression of studied factors and clinicopathological variables were evaluated by the Person χ^2^ test. The Kaplan -Meier method and the log -rank test were employed to estimate and compare survival rate. For growth rate or inhibition rate analyses Student-*t* test was applied. All calculation was performed by usage of the SPSS 16.0 statistical software package (SPSS, Chicago, IL), and *p*≤0.05 was considered as statistical significance.

## Results

### Expression of Notch family in the cell lines

**Figure 1:**
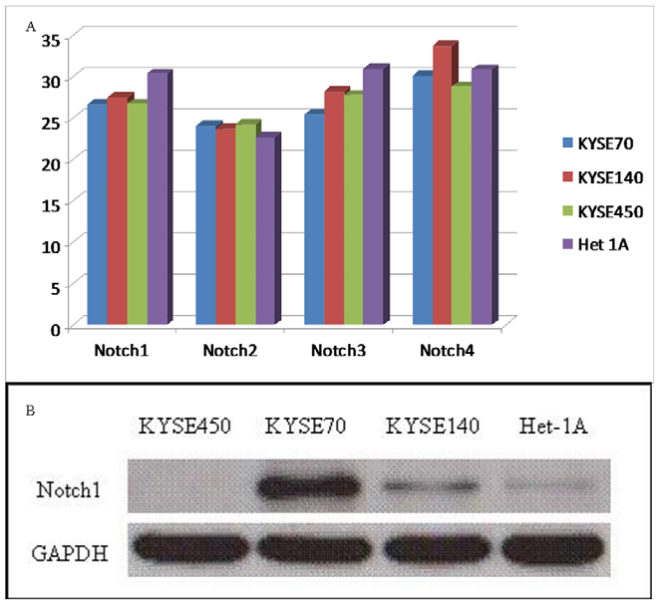
Quantitative RT-PCR of Notch family in the squamous esophageal cancer cell lines KYSE70, KYSE140 and KYSE450 and the virus transformed normal squamous esophageal epithelial cell line Het-1A (A). Each PCR was performed twice with almost identical values. Notch1 protein expression was examined by Western blotting (B), showing strong Notch1 in KYSE70 cells, negative in KYSE450 cells and weak positive in both KYSE140 and Het-1A cells. Conventional RT-PCR shows rather the same intensity PCR bands for both KYSE70 and KYSE450 cells with the two primer pairs (C).

Quantitative RT-PCR analyses revealed rather equal amount of Notch2 in the four cell lines. Although Notch4 RNA expression in two cancer cell lines was slightly higher than the Het-1A cell line, the KYSE70 cell line expressed lower level of Notch4, and in general the Nocth4 expression in these four cell lines was low. Higher levels of both Notch1 and Notch3 RNA expression in the cancer cell lines than that in the Het-1A cell line were repeatedly verified (Figure 1A). Equal Notch2 expression and weak Notch4 expression in these four cell lines did not encourage further analyses of these two factors. Due to the fact that Notch3 was rather clear with a role of squamous differentiation in esophageal epithelial cells [21], [22], Notch1 was chosen for further functional and clinicopathological studies in this project. Western blotting revealed that KYSE70 expressed high level Notch1, KYSE140 and Het-1A were weakly positive for Notch1 while KYSE450 was negative for Notch1 (Figure 1B). In order to better study the function of Notch1, the strong Notch1 positive cell line KYSE70 and the Notch1 negative cell line KYSE450 were further analyzed. Conventional RT-PCR with the two pairs of Notch1 specific primers confirmed rather equal intensity of PCR products in both KYSE70 and KYSE450 cells (Figure 1C). Sequencing of the PCR products disclosed neither mutation nor deletion (Figure S1 and Figure S2).

### Growth effect of hypoxia on the KYSE450 and KYSE70 cell lines

**Figure 2:**
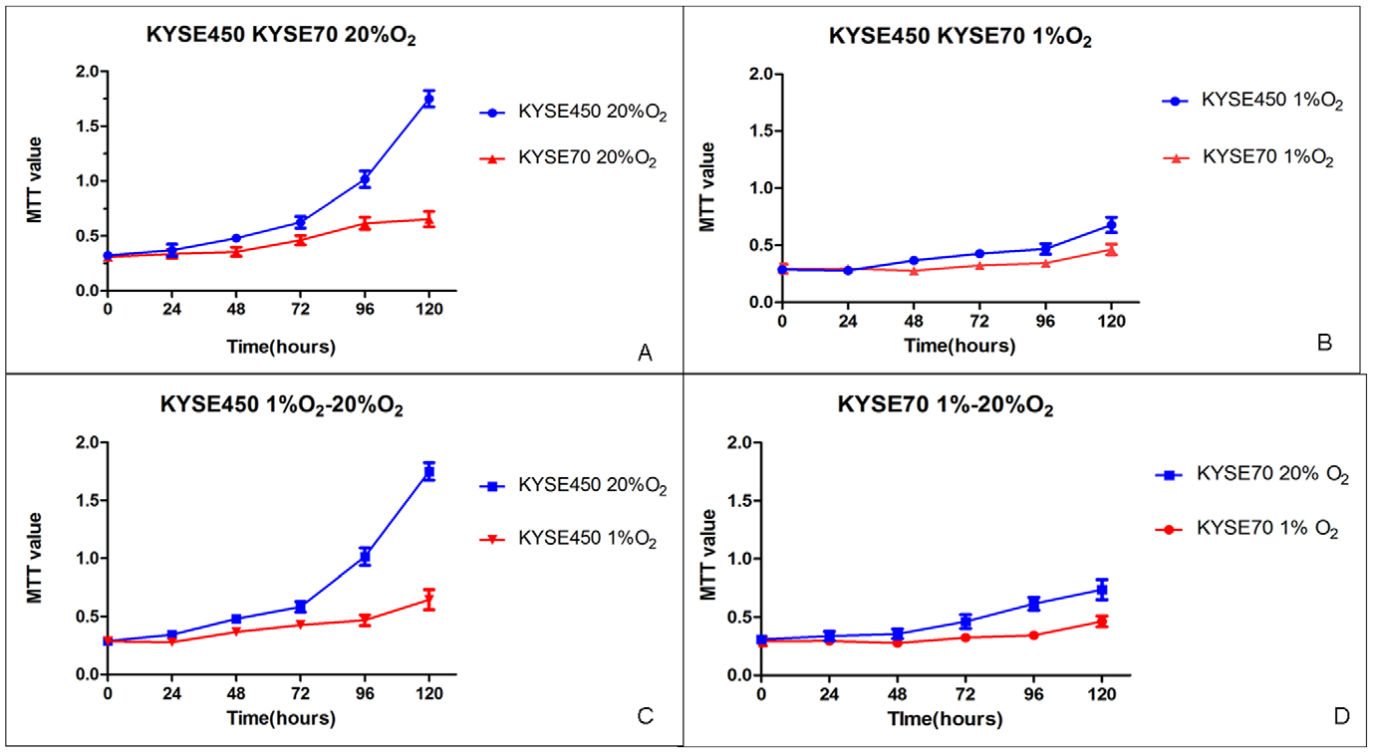
Cell growth curves show that both cell lines are growth-inhibited under 1% O^2^ than under 20% O^2^. KYSE70 cells tolerate hypoxia better than KYSE450 cells.

The cell growth influence of hypoxia (1% O2), in comparison to the cells cultivated in normoxia (20% O2), was studied with MTT assay. As shown in Figure 2A and B, KYSE450 cells grow faster than KYSE70 cells under normoxia condition. However, upon placed in 1% O2, the growth difference of these two cell lines is less prominent. The growth difference of these two cell lines in normoxia and hypoxia is displayed in Figure 2C, where apparent growth difference of KYSE450 cells is shown while in Figure 2D the growth difference in KYSE70 cells is not prominent. Statistical analysis of these two groups of data in Figure 2C and Figure 2D reveals significant difference (P<0.001).

### Notch1 is hypoxia inducible in the KYSE70 cell line

**Figure 3:**
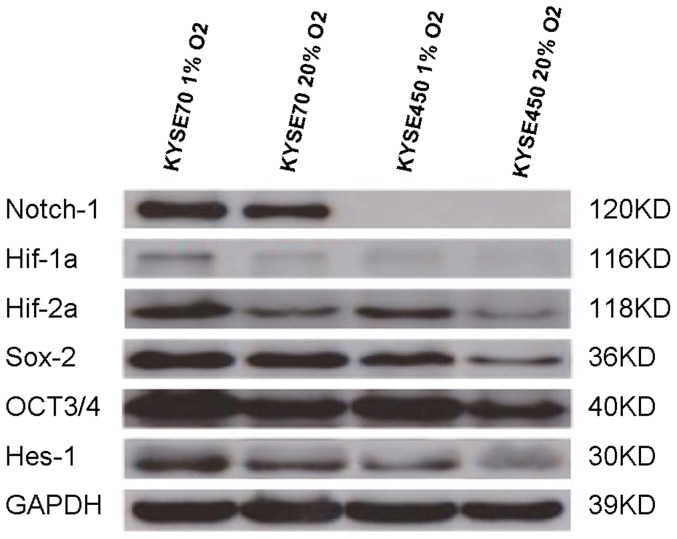
Hypoxia induction of Notch1 and other stemness factors *in vitro*. Notch1 is absent in the KYSE450 cells, but positive in KYSE70 cells under normoxia and even high level of Notch1 expression in these KYSE70 cells under hypoxia is shown. There are weak Hif-1α expression under normoxia and weak Hif-1α induction under hypoxia in KYSE450 cells. Comparably higher levels of expression and Hif-1α induction in KYSE70 cells are shown. Positivity and hypoxia induction of Hif-2α, Sox2, Oct3/4 and Hes-1 in KYSE450 cells and relatively stronger positivity and higher levels of hypoxia induction of these factors in KYSE70 cells are displayed.

To analyze the effect of hypoxia on cell stemness in these cells the expressions of Oct3/4, Sox2, Notch1 and Hes-1 were measured by Western blotting, in addition to the expressions of Hif-1α and Hif-2α. As shown in Figure 3, the cells cultivated in 1% O2 for 48 hrs revealed higher levels of Oct3/4, Sox2 and Hes-1 expressions, compared to the cells cultivated in normoxia for the same time period. Elevated Hif2α expressions were seen in both cell lines. For Hif-1α, apparently lower level expression in the KYSE450 cell line in 20% O^2^ was repeatedly observed and the induction of this factor in 1% O^2^ was not apparent compared to KYSE70 cell line in which there was hypoxic induction of Hif-1α expression. Again as shown in Figure 3, Notch1 was highly expressed in the KYSE70 cell line in normoxia and its expression was even induced in hypoxia in a greater level. However no expression in normoxia and no hypoxia induction of this factor could be detected in the KYSE450 cell line.

### KYSE70 cell line is more 5-FU resistant

**Figure 4:**
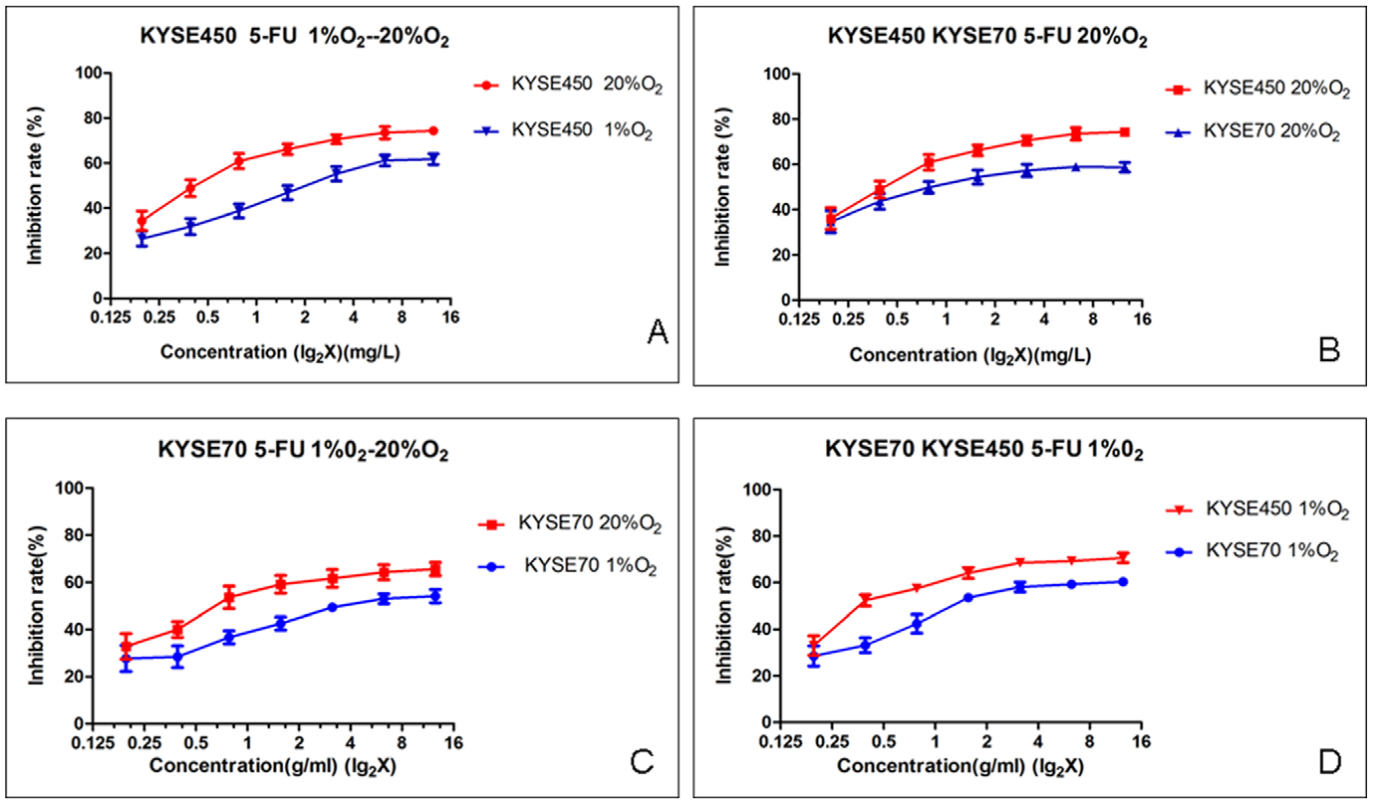
KYSE70 cells tolerate higher concentrations of 5-FU than KYSE450 cells under normoxia, and such tolerance difference is even significantly apparent under hypoxia.

Since Notch1 was indicated in promotion of tumor invasion, metastasis and EMT [2], [20], a feature related to cancer stem cells, and Notch1 expressed in one cell line, but not in another, we were attempted to assess whether these two cell lines had different 5-FU response. Therefore dose dependent 5-Fu growth inhibition experiments were performed in the cells with 48 hrs 5-Fu exposure. As shown in Figure 4A, the Notch1 negative cell line KYSE450 cells show a dose dependent sensitivity (growth inhibition) cultivated in 20% O^2^, and such inhibition could be reduced in the cells cultivated in 1% O^2^. The Notch1 positive KYSE70 cells were less sensitive to the 5-FU treatment in normoxia, compared to KYSE450 cells under the same condition (Figure 4B). If the KYSE70 cells were cultivated in 1% O^2^ compared to the KYSE70 cells in mormoxia, significantly higher 5-FU resistance was seen (p<0.001, Figure 4C). Figure 4D shows significantly less 5-FU growth inhibition in KYSE70 cells than in KYSE450 cells under hypoxia condition (P<0.001).

### Notch1 expression is linked to 5-FU resistance

**Figure 5:**
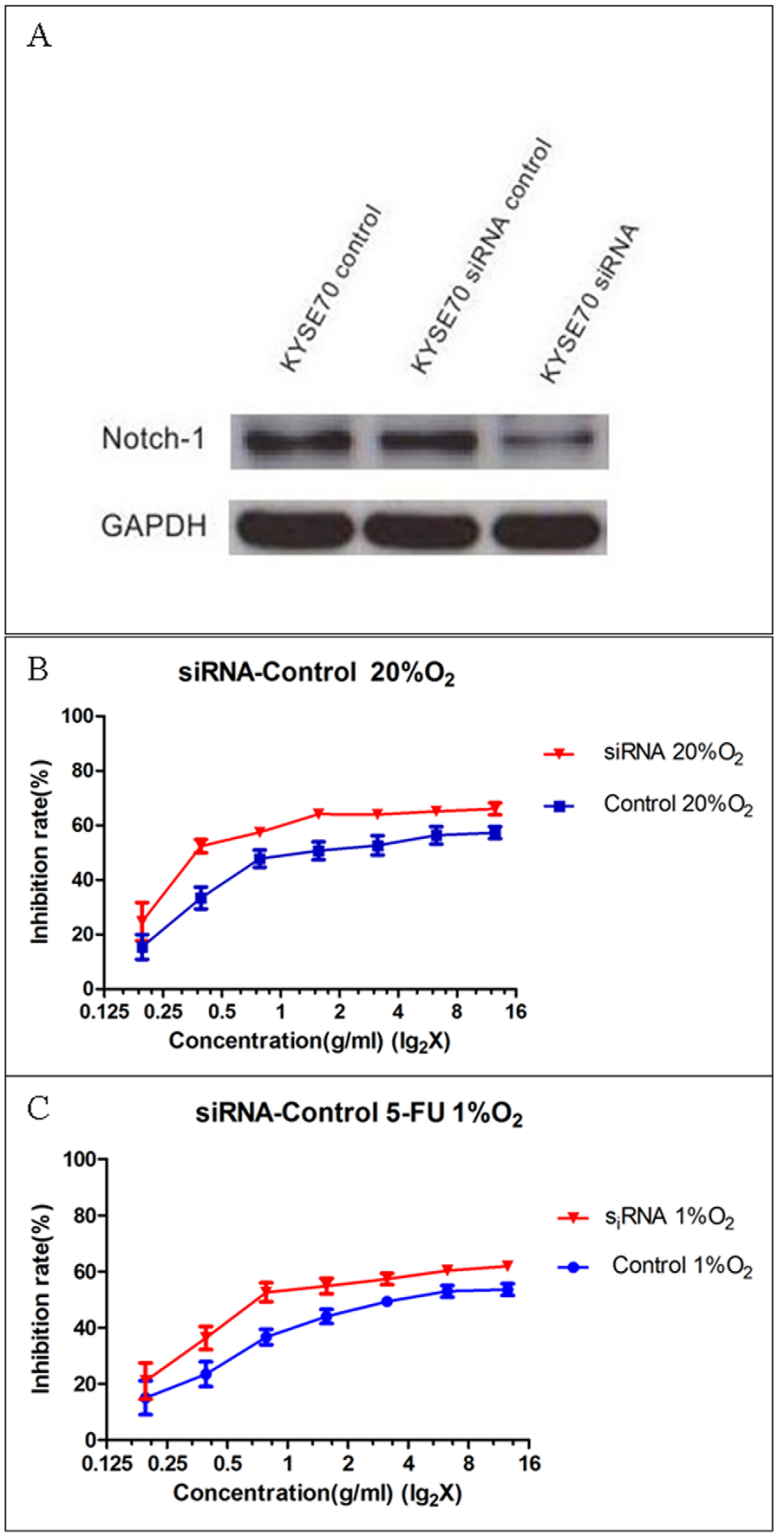
Notch1 siRNA knockdown validation in KYSE70 cells (5A). KYSE70 control means blank KYSE70 cells without any treatment. KYSE70 siRNA control means KYSE70 cells transfected with the non-specific siRNA. KYSE70 siRNA means KYSE70 cells transfected with the specific siRNA for Notch1. Notch1 siRNA knockdown in the KYSE70 cells significantly increases their 5-FU chemosensitivity both in normoxia (B) and hypoxia (C).

The next step we asked whether that was Notch1 playing a role in such 5-FU resistance difference. To answer this question, Notch1 knockdown was performed in this KYSE70 cell line with a siRNA technology. As shown in Figure 5A, 80% of the Notch1 protein expression could be blocked by this Notch1 siRNA technology 48 hrs after siRNA transfection. Therefore 5-FU dose dependent cell growth inhibition experiments were performed in these cells 48 hrs after Notch1 specific or non-specific siRNA treatments. In normoxia condition elevated levels of 5-FU sensitivity were repeatedly shown in the Notch1 specific siRNA treated cells (Figure 5B), compared to the Notch1 non-specific siRNA treated control cells (p = 0.004). Similarly cultivated in 1% O^2^ significantly higher levels of 5-FU sensitivity were observed in the Notch1 specific siRNA treated cells (Figure 5C, p = 0.004).

### Notch1 expression is significantly associated with poor clinical outcome

**Figure 6:**
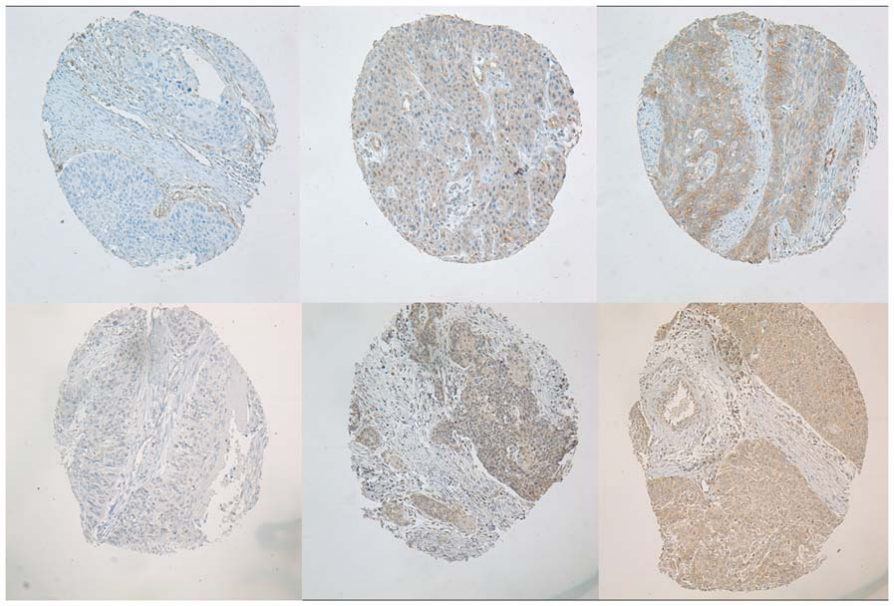
Variable Notch1 and Hes1 expressions revealed in the tissue microarray sections. Upper panel is for Notch1 expression and lower panel is for Hes-1 expression. All images were taken at 200 x.

**Figure 7:**
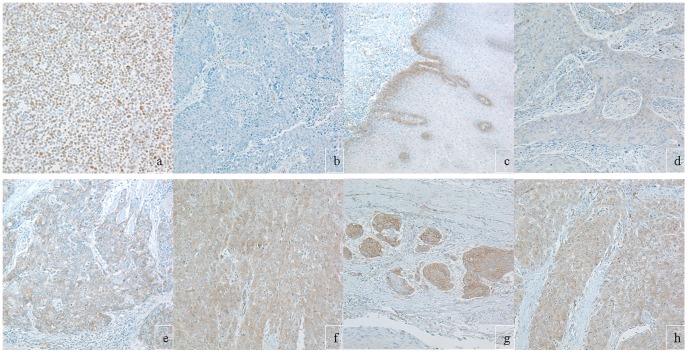
Immunohistochemical results of Notch1. (a) KYSE70 cells with mainly cytoplasmic and membrane staining in most of the cells and a few cells with nuclear staining. (b) a negative Notch1 tumor. (c) normal esophagus epithelial basal cells with cytoplasmic and membrane staining. (d, e and f) represent weak, moderate and strong Notch1 expressions in different squamous cell carcinomas, respectively. In addition, strong Notch1 expression is shown within vascular structures (g) and an infiltration front (h). All images were taken at 200 x.

**Figure 8:**
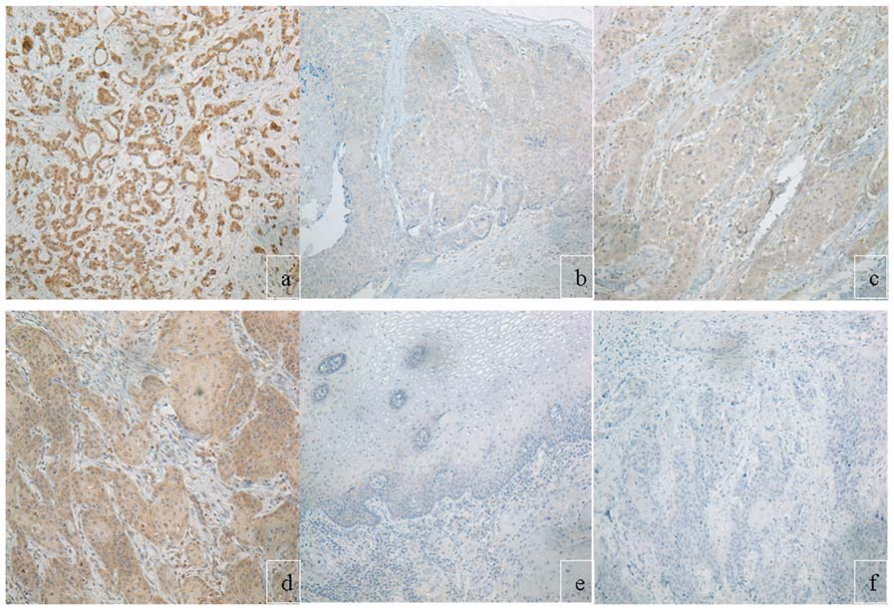
Immunohistochemical results of Hes1. (a) positive control of known Hes1 positive breast carcinoma. (b, c and d) represent weak, moderate and strong Hes1 expressions in squamous cell carcinomas, respectively. (e) negative Hes1 in a normal esophagus epithelium. (f) negative Hes-1 in a squamous cell carcinoma. All images were taken at 200 x.

**Figure 9:**
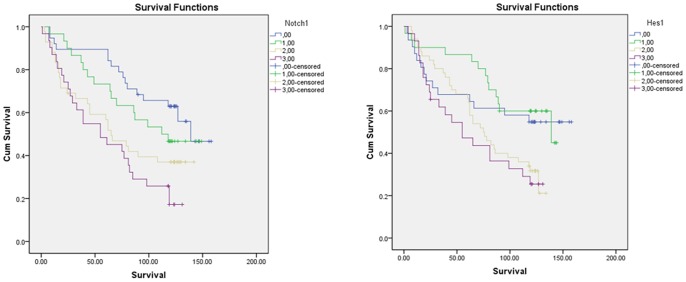
Overall survival curves. Significantly shorter overall survival (in month) is shown for the patients with higher levels of Notch1 (p<0.001), but Hes-1 expression is not correlated to survival (p = 0.442).

Immunohistochemical screening of the tissue microarray sections revealed variable Notch1 (Figure 6, upper panel) and Her-1 expressions (Figure 6, lower panel). To closely examine their expression status in these samples, large paraffin sections were applied for additional immunohistochemistry. As shown in Figure 7, Notch1 protein expression could be revealed in the ten normal epithelia adjacent to tumors. In these normal epithelia positive reaction was mainly localized in the membrane and cytoplasm of the basal cells. In the tumor samples, rather homogeneous staining could be seen in the poorly differentiated tumors, while the well differentiated tumors were mainly positive in their basal layers of the tumor nests, and some tumors were negative for Notch1 expression. The KYSE70 cells were always positive for this antibody (Figure 7a). In addition vascular invasion (Figure 7g) and infiltration front (Figure 7h) were observed strongly positive for the Notch1 immunostaining as well. Cytoplasmic HES1 immunoreaction could be observed in tumor cells, but its expression in the basal cells of normal epithelium was absent (Figure 8). Of the 156 tumors 40 (25.6%) were negative, 32 (20.5%) were weakly positive, 47 (30.1%) were moderately positive and 37 (23.7%) were strong positive for Notch1. For Hes-1 expression, 33 (21.2%), 36 (23.1%), 53 (34.0%) and 34 (21.8%) of the 156 tumors were negative, weakly positive, moderately positive and strong positive, respectively (Table 1, p = 0.05). Higher levels of Notch1 expression in these tumors were significantly associated with higher pathological grade (p = 0.003) and clinical stage (p = 0.031), but not associated with tumor size (p = 0.426). However Hes-1 expression was neither correlated with tumor grade (p = 0.237), nor with clinical stage (p = 0.397). Overall survival analyses of the patients with 10 years follow-up show a significantly shorter clinical outcome for patients with higher levels of Notch1 expression(Figure 9, p = 0.001), but not with Hes-1 expression.

**Table 1:** Notch1 and Hes1 Crosstabulation.

		Hes1 immunohistochemical score				
		Negative	1	2	3	Total
Notch1Immunohistochemical score	Negative	11	15	7	7	40
	1	6	11	10	6	33
	2	9	8	19	11	47
	3	7	3	17	10	37
Total		33	37	53	34	157

## Discussion

We have confirmed the Notch1 expression in the morphologically aggressive KYSE70 human esophagus squamous cancer cell line by both Q-PCR and Western blotting, and its expression is inducible in hypoxia condition. However, the Notch1 protein expression was confirmed negative in the KYSE450 cells and weakly positive in the KYSE140 cells, although Notch1 RNA expressed in a similar level in these three cancer cell lines. Sequencing of the RT-PCR products of Notch1 in both the KYSE70 and KYSE450 cells with two Notch1 specific primer pairs disclosed neither mutation nor deletion, indicating possible post-transcriptional regulations of Notch1 in the KYSE450 cells. Further study revealed that the KYSE70 cell line tolerated hypoxia treatment better than the KYSE450 cell line as shown in Figure 2.

In consistent with our previous study in prostate cancer cells with greater stemness in response to hypoxia[26], both KYSE70 and KYSE450 esophagus cancer cell lines were responding to hypoxia by upregulation of the stemness factors Oct3/4 and Sox2. Next, we decided to ask whether the cells' 5-FU chemotherapy sensitivity was reduced in hypoxia condition. Our results showed that both cell lines were more resistant to the 5-FU treatment in hypoxia than in normoxia. However, we discovered that the KYSE70 cells showed significantly higher 5-FU resistance under both normoxia and hypoxia than the KYSE450 cells did. This led us to speculate whether Notch1 might have some role in this 5-FU resistance difference, since the major difference of these two cell lines is their Notch1 expression. Although other protein expression differences in these two cell lines like Hif-1α and Hes-1 were discovered, only Notch1 expression was verified always negative in the KYSE450 cells, and always strong positive in the KYSE70 cells. Therefore we performed additional 5-FU sensitivity experiments in the KYSE70 cells in which Notch1 gene expression was knocked down. It was repeatedly shown correspondingly reduced levels of 5-FU resistance or increasing levels of 5-FU sensitivity in these Notch1 gene blocked KYSE70 cells (Figure 5). These results strongly indicate a role of Notch1 in chemotherapy resistance [27], [28], [29], [30], a common feature of cancer stem cells. Most commonly if a tumor is resistant to chemotherapy it may also be resistant to radiotherapy, also a common feature linked to cancer stem cells[31]. If that is true there might be clinical significance of Notch1 expression in tumor samples. To answer this question, we ran an immunohistochemical study of Notch1 and Hes-1 in a series of human squamous cell carcinomas collected in a high-risk area in China. The clinical information was obtained through a Sino-Norwegian esophageal cancer collaboration project and both parties verified the quality of samples. Through screening of a tissue microarray of the material we discovered that Notch1 and Hes-1 were variably expressed in these tumors. To obtain rather accurate estimation we carried out immunohistochemistry with large sections for all of these samples. We found that Notch1 was expressed in the basal layers of normal esophagus epithelia while in tumors, if it was positive, rather homogeneously expression was seen, except in the well differentiated tumors where mainly basal layers of the tumor nests were positive. Strong Notch1 expression was also seen in the infiltration fronts and the vascular invasions, phenomena indicating cellś aggressiveness. Clinical pathological analyses revealed its significant associations with higher pathological grade and poorer overall survival. These observations are largely in line with the reports in leukemia[32], [33], gastric cancer [34] and colorectal carcinomas[31], [35], [36], [37], [38] where Notch1 was linked to an oncogenic role.

Gustavsson et al [39] and Zheng X et al[40] have documented that hypoxia blocks neuronal and myogenic differentiation in a Notch-dependent manner and Notch intracellular domain interacts with Hif-1α so that Hif-1α is recruited to Notch-responsive promoters upon Notch activation under hypoxic conditions. Varnum-Fun et al[41], Pistollato et al[42] and Main et al[43] also reported similar findings. In our present study the KYSE450 cells were almost negative for Hif-1α protein expression, and its expression was even not inducible in hypoxia. In parallel with these findings, Notch1 expression in these cells was also not detectable, contrasting to the KYSE70 cells. Morphologically these two cell lines still kept their original differentiation feature. If the cells in culture were close to confluent and collected with rubber scratch for cytoblock and section preparation, the KYSE450 cells under microscopy revealed epithelial-like structure, a well differentiation feature; while the KYSE70 cells were rather cellular, a poor differentiation indication. It will be the next step to study whether it is the higher levels of the stemness-related factors of Oct3/4, Sox2 and Notch1 in this cell line together determining the poor differentiation status. Indeed the KYSE70 cells were repeatedly shown in our lab containing about 1% side population (SP) cells, a feature of stem cells, while SP cells in the KYSE450 cells were never detected (data not shown). Notch signaling pathway has been found to play a central role in induction of epithelial-mesenchymal transition (EMT), also a feature of cancer stem cells [44], [45].

However these findings disagree with those studies where tumor suppressor properties of Notch1 are suggested. Agrawal et al [20] discovered in a whole exome sequencing study of a series 32 primary head and neck squamous cell tumors that nearly 40% of the 28 mutations identified in Notch1 were predicted to truncate the gene product, and they suggest that Notch1 may function as a tumor suppressor gene rather than an oncogene in this tumor type. Similar finding was also reported by Stransky et al [46]. Using a tissue-specific Notch1 knockout approach in a mouse model Nicolas et al [13] found that ablation of Notch1 resulted in epidermal and corneal hyperplasia followed by the development of skin tumors and facilitated chemical-induced skin carcinogenesis. Even in esophagus studies, contradictory results exist. Lu et al[17] in a study of Notch signaling in a squamous cancer cell line indicates its G1/G0 maintenance, which may suggest its function as stemness maintaining factor. However, other reports indicate its function in esophageal epithelial cell differentiation [22], [34]. Therefore, Notch1 expression in tumors may function as a double-edged sword[47], most probably depending on the whole complex a given tumor faces.

We speculate the following possibility for Notch1 expression in these tumors: Notch1 may play an important role in carcinogenic process as indicated in different studies [5], [48]. However, double edge function of Notch1 do exist, not only because there are strong lines of evidence as suppressor[47], [49], but also because about one fourth of the tumors in our study were negative for Notch1 expression; Functional mutation of Notch1 may trigger Notch1 overexpression in the rest cells of tumor by a till now unexplored mechanism, so that both functional mutation and overexpression of Notch1 may exist in the same tumors in different phases of tumor development. The overexpression of Notch1 may endorse the tumor cells with higher stemness, more therapy-resistance and strong potential in invasion and metastasis as partly indicated in our study and partly in literature, while functional mutation of Notch1 may play an important role in tumor initiation. However, the Notch1 negative tumors may in general be less aggressive as verified in our current study, most probably due to lack of Notch1 function. That the Hes-1 expression in these tumors was not associated with poor survival as Notch1 did may indicate aberrant Notch1 signaling which merits further studies.

In summary, the aggressive KYSE70 human esophageal squamous cell carcinoma cell line is positive for Notch1 expression, and such expression is hypoxia-inducible; the less aggressive KYSE450 human squamous cell carcinoma cell line is negative for Notch1 and the Notch1 expression in this cell line is not inducible; the KYSE70 cell line is more 5-FU resistant than the KYSE450 cell line, and such 5-FU resistance is correlated to Notch1 expression verified by Notch1 siRNA experiment; Notch1 expression is positive in the basal layer of human esophagus epithelia, and its expression in squamous cell carcinomas is significantly correlated to higher pathological grade and shorter survival. The molecular mechanism behind these findings warrants additional studies.

## Supporting Information

Figure S1 **Forward primer sequencing result of Notch1 in KYSE 70 human esophageal squamous cell carcinoma cells (from 5'to 3'):GGCATGGTGCCGAACCAATACAACCCTCTGCGGGGGAGTGTGGCACCAGGCCCCCTGAGCACACAGGCCCCCTCCCTGCAGCATGGCATGGTAGGCCCGCTGCACAGTAGCCTTGCTGCCAGCGCCCTGTCCCAGATGATGAGCTACCAGGGCCTGCCCAGCACCCGGCTGGCCACCCAGCCTCACCTGGTGCAGACCCAGCAGGTGCAGCCACAAAACTTACAGATGCAGCAGCAGAACCTGCAGCCAGCAAACA.**
(TIF)

Figure S2 **Reward primer sequencing result of Notch1 in KYSE 70 human esophageal squamous cell carcinoma cells (from 5'to 3'):GGTCCACCAGTTTGAATGGTCAATGCGAGTGGCTGTCCCGGCTGCAGAGCGGCATGGTGCCGAACCAATACAACCCTCTGCGGGGGAGTGTGGCACCAGGCCCCCTGAGCACACAGGCCCCCTCCCTGCAGCATGGCATGGTAGGCCCGCTGCACAGTAGCCTTGCTGCCAGCGCCCTGTCCCAGATGATGAGCTACCAGGGCCTGCCCAGCACCCGGCTGGCCACCCAGCCTCACCTGGTGCAGACCCAGCAGGTGCAGCCACAAAAA.**
(TIF)
