# Supplementary figures and images for: Notch1 Is a 5-Fluorouracil Resistant and Poor Survival Marker in Human Esophagus Squamous Cell Carcinomas
Source: PLoS One. 2013 Feb 7;8(2):e56141. doi: 10.1371/journal.pone.0056141 (PMC3567068; doi:10.1371/journal.pone.0056141)

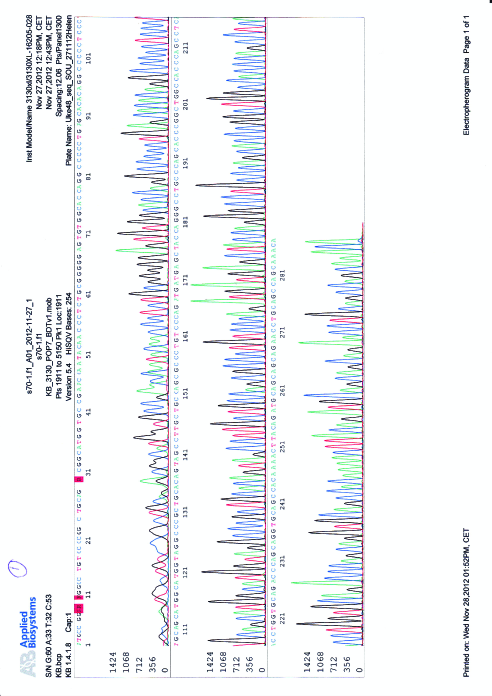

Supplement: Figure S1 — Forward primer sequencing result of Notch1 in KYSE 70 human esophageal squamous cell carcinoma cells (from 5'to 3'):GGCATGGTGCCGAACCAATACAACCCTCTGCGGGGGAGTGTGGCACCAGGCCCCCTGAGCACACAGGCCCCCTCCCTGCAGCATGGCATGGTAGGCCCGCTGCACAGTAGCCTTGCTGCCAGCGCCCTGTCCCAGATGATGAGCTACCAGGGCCTGCCCAGCACCCGGCTGGCCACCCAGCCTCACCTGGTGCAGACCCAGCAGGTGCAGCCACAAAACTTACAGATGCAGCAGCAGAACCTGCAGCCAGCAAACA. (TIF) [file pone.0056141.s001.tif]

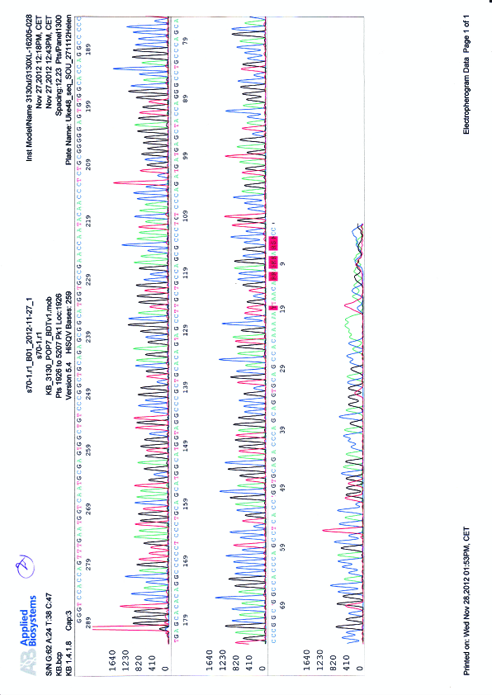

Supplement: Figure S2 — Reward primer sequencing result of Notch1 in KYSE 70 human esophageal squamous cell carcinoma cells (from 5'to 3'):GGTCCACCAGTTTGAATGGTCAATGCGAGTGGCTGTCCCGGCTGCAGAGCGGCATGGTGCCGAACCAATACAACCCTCTGCGGGGGAGTGTGGCACCAGGCCCCCTGAGCACACAGGCCCCCTCCCTGCAGCATGGCATGGTAGGCCCGCTGCACAGTAGCCTTGCTGCCAGCGCCCTGTCCCAGATGATGAGCTACCAGGGCCTGCCCAGCACCCGGCTGGCCACCCAGCCTCACCTGGTGCAGACCCAGCAGGTGCAGCCACAAAAA. (TIF) [file pone.0056141.s002.tif]
